# Traditional Chinese and Western medicine-integrated risk prediction models for coronary heart disease: a systematic review and meta-analysis

**DOI:** 10.3389/fcvm.2026.1921708

**Published:** 2026-09-25

**Authors:** Chenggong Xie, Huiying Zhuang, Zhengquan Du, Yingyi Zhou, Hao Liang, Bin Wang

**Affiliations:** 1 Institute of Information on Traditional Chinese Medicine, China Academy of Chinese Medical Sciences, Beijing, China; 2 School of Acupuncture and Tui-na and Rehabilitation, Hunan University of Chinese Medicine, Changsha, China; 3 Academy of Chinese Medical Sciences, Hunan University of Chinese Medicine, Changsha, China

**Keywords:** coronary heart disease, meta-analysis, risk prediction models, Traditional Chinese Medicine, Traditional Chinese and Western medicine-integrated

## Abstract

**Background:**

Coronary heart disease (CHD) remains a leading global cause of mortality, necessitating improved early risk prediction. Traditional Chinese Medicine (TCM) may provide complementary diagnostic information for models based on Western medicine (WM), but the performance and certainty of TCM-WM integrated models remain unclear.

**Methods:**

This systematic review and meta-analysis evaluated TCM-WM integrated risk prediction models for CHD. Sixteen studies were included; diagnostic-accuracy outcomes were synthesized from 12 studies and odds ratios (ORs) from 7 studies. Heterogeneity, risk of bias, subgroup effects, and meta-regression findings were assessed.

**Results:**

The pooled sensitivity was 0.79 (95% CI: 0.62–0.89; I² = 98.17%), specificity was 0.87 (95% CI: 0.76–0.94; I² = 99.39%), and summary AUC was 0.90 (95% CI: 0.88–0.93). The pooled OR was 1.80 (95% CI: 1.40–2.30; I² = 95.6%). Twelve of 16 studies had high or unclear overall risk of bias, and only 5 studies reported external validation. Although subgroup analyses suggested possible differences according to the number of TCM predictors, these analyses were exploratory and did not resolve the extreme between-study heterogeneity.

**Conclusions:**

TCM-WM integrated models yielded favorable average discrimination estimates, but extreme heterogeneity, methodological limitations, and limited external validation substantially reduce certainty in the pooled estimates. The findings should therefore be considered hypothesis-generating and do not establish reliable performance or clinical benefit across settings. Multicenter prospective validation, standardized TCM definitions, and transparent reporting according to TRIPOD and PROBAST are required before clinical implementation.

**Systematic Review Registration:**

https://www.crd.york.ac.uk/PROSPERO/view/CRD420250652605, CRD420250652605.

## 1 Introduction

Coronary heart disease (CHD) remains a leading global cause of morbidity and mortality, accounting for 32% of cardiovascular deaths worldwide (1). In 2019, the prevalence of CVD in China was 120 million, representing a 140.02% increase since 1990 (2). These trends highlight the need for improved risk prediction to enable early intervention.

Data-driven prediction models, particularly those utilizing electronic health records, are increasingly influencing clinical decision-making (3). However, current Western medicine (WM) risk models, such as the Framingham Risk Score (FRS) and ACC/AHA pooled cohort equations (PCEs), have limitations (4, 5). Current models overemphasize biomarkers while overlooking psychosocial and holistic determinants—non-traditional risk factors demonstrated to enhance diagnostic efficacy (6). TCM’s four diagnostic modalities (inspection, auscultation-olfaction, inquiry, and palpation) show particular promise as unconventional predictors, exhibiting excellent diagnostic performance in prediction models (7).

Integrating WM’s quantitative metrics with traditional Chinese medicine’s four diagnostic methods may enhance CHD risk prediction (8). However, challenges remain. Outcome assessment in integrative medicine remains challenging, primarily due to fundamental conceptual disparities between WM’s disease-oriented framework and TCM’s syndrome-differentiation paradigm (9). Furthermore, distinct biological bases underlie different TCM syndromes of the same disease, adding complexity to integrated diagnosis (10). This warrants systematic evaluation of integrative CHD prediction models to address methodological and data challenges.

This study systematically evaluates the reported performance of integrated CHD risk models (including OR, AUC, sensitivity, and specificity) and explores the contribution of TCM elements. The synthesis is intended to characterize the current evidence, identify sources of uncertainty and heterogeneity, and define priorities for model development and validation rather than to support immediate clinical implementation.

## 2 Methods

This systematic review adheres to PRISMA-DTA guidelines and prediction model performance guidelines (PROSPERO registration: CRD420250652605) (11, 12). As all analyses utilized aggregated data from published studies, neither ethical approval nor individual patient consent was required.

### 2.1 Search strategy

Literature searches spanned PubMed, Web of Science, Embase, Google Scholar, China National Knowledge Infrastructure (CNKI), Wanfang, and VIP databases from inception through 3 March 2025, using optimized subject headings and keywords tailored to each platform. Core search terms included “Coronary Artery Disease”, “TCM”, “Syndrome Differentiation”, “Risk Prediction Model”, “Statistical Model”, and “Clinical Prediction”. Full search strategy for each database are provided in Supplementary Table 1.

We employed the PICOTS (Population, Intervention, Comparator, Outcome, Timing, Setting) framework as a guiding structure for defining inclusion and exclusion criteria. Outlined below are the specific principles of the PICOTS framework:

P (Population): The target population comprised adults with a confirmed diagnosis of CHD, established according to standardized diagnostic criteria outlined in authoritative WM guidelines (e.g., the ESC or ACC/AHA guidelines), and/or syndrome differentiation based on principles defined in official TCM diagnostic frameworks or national expert consensus.

I (Intervention model): We examined risk prediction models explicitly designed to estimate CHD risk by integrating variables from both Western clinical diagnostics (e.g., lipid levels, ECG findings, imaging) and TCM assessment (e.g., pulse diagnosis and tongue features classification), with each model incorporating at least five predictive factors.

C (Comparator): No direct comparison with alternative models was required.
O (Outcome): The primary outcome was the predicted risk of incident CHD events (e.g., myocardial infarction, unstable angina), validated through measures of discrimination. Studies addressing unrelated outcomes (e.g., stroke, heart failure) were excluded.
T (Timing): The timing of prediction focused on baseline assessment prior to acute events or therapeutic interventions, using clinical and TCM-derived data available at the initial point of evaluation.
S (Setting): Eligible studies were conducted in clinical or community settings where both TCM and WM were used concurrently, with the intent of informing individualized risk stratification and preventive decision-making in clinic.

### 2.2 Study selection

Two independent reviewers (D.Z.Q. and Z.Y.Y.) conducted screening using predefined criteria.

Inclusion criteria:

1) Development/validation of CHD risk models integrating TCM (diagnosis or treatment) and Western medicine (biomarkers/imaging),
2) Primary study objective including diagnostic identification of CHD,
3) Reporting of ≥1 performance metric (OR, AUC, sensitivity, specificity, or calibration).

Exclusion criteria:

1) Models using Western medicine-only or TCM-only predictors,
2) Studies lacking reported performance metrics,
3) Sample sizes <100 patients or <3 risk predictors,
4) Non-CHD cardiovascular studies (e.g., stroke-only models),
5) Non-English/Chinese publications without validated translations.

### 2.3 Data extraction and quality assessment

Predefined data elements were systematically extracted, including study metadata (author, year, country), study design, participant characteristics (age range, clinical features), data sources, disease outcomes, case proportions and performance metrics (OR, AUC, sensitivity and specificity).

In this study, predictors were classified as TCM-related or WM-related according to their theoretical basis and diagnostic role. TCM-related predictors included TCM patterns, syndrome elements, constitution, tongue and pulse characteristics, and quantitative parameters derived from objective pulse assessment. Pulse-wave-derived parameters, including pulse wave velocity (PWV), were classified as TCM-related when used as quantitative extensions of pulse-related assessment within an integrative diagnostic framework. WM-related predictors included conventional cardiovascular risk factors, medical history, laboratory tests, electrocardiogram (ECG), imaging findings, and other biomedical measurements.

To assess the quality and potential bias risk of the included studies, we employed the Prediction Model Risk of Bias Assessment Tool (PROBAST). This tool facilitated the evaluation of bias and concerns regarding the applicability of the included studies through the systematic application of the PROBAST checklist.

PROBAST, with two independent reviewers (D.Z.Q. and Z.Y.Y.) assessing each study. The tool’s four domains (participants, predictors, outcome, and analysis) were rigorously applied to evaluate both bias risk and clinical applicability.

Reviewers scored each domain using standardized signaling questions (*n* = 20), with responses categorized as:

Low risk (“yes”/“probably yes”)
High risk (“no”/“probably no”/unreported)
Final risk classification followed PROBAST guidelines:
Low overall risk: All domains low risk
High overall risk: ≥1 domain high risk

### 2.4 Data synthesis

Prediction model performance was quantitatively synthesized through confusion matrix parameters (true positive (TP), false positive (FP), false negative (FN), true negative (TN)) and OR value. Where possible, unreported values were calculated using performance metrics (sensitivity and specificity); studies with insufficient data for derivation were excluded from quantitative synthesis.

To explore potential sources of substantial heterogeneity, subgroup analyses and meta-regression were conducted based on two predefined dimensions: the degree of standardization in TCM methodology and the number of TCM-related variables incorporated into the models.

For TCM standardization, studies were categorized as highly standardized if they explicitly referenced recognized TCM clinical guidelines or authoritative literature in defining diagnostic or therapeutic principles. In contrast, studies lacking such references were classified as low standardization.

For the number of TCM predictors, models were classified as univariate when only a single type of TCM element—such as tongue diagnosis or pulse characteristics—was included, based on categorizations from standard TCM. When more than one distinct category of TCM factors was incorporated, the model was defined as multivariate. Notably, multiple tongue-related variables (e.g., tongue color and coating) were considered as a single category (tongue features), and therefore counted as univariate.

To further explore potential TCM and non-TCM sources of heterogeneity, additional subgroup analyses were conducted based on TCM predictors and WM predictors.

### 2.5 Statistical analysis

Bivariate and hierarchical summary receiver operating characteristic (HSROC) modeling jointly estimated diagnostic accuracy parameters (OR, AUC, sensitivity, specificity). Predefined subgroup analyses and meta-regression were implemented using R 4.2.1 and Stata 14.1.

Statistical heterogeneity across studies was evaluated using the Cochran’s *Q* test and the I² statistic, which quantifies the proportion of total variability attributable to between-study differences rather than chance. An I² value below 50% was interpreted as low heterogeneity, for which a fixed-effects model was applied. In contrast, substantial heterogeneity (I²> 50%) prompted the use of a random-effects model to account for inter-study.

Sensitivity analyses were conducted using a leave-one-out approach, in which each study was sequentially excluded and the pooled estimates were recalculated. For diagnostic-accuracy outcomes, changes in pooled sensitivity, specificity, and summary AUC were examined; for the OR analysis, changes in the pooled OR were assessed.

## 3 Results

### 3.1 Study selection

Study selection development involved D.Z.Q., Z.Y.Y., and X.C.G. (principal investigator). From 7691 initially identified records, title and abstract screening yielded 63 studies for full-text review, with 15 ultimately meeting inclusion criteria for quality assessment and meta-analysis (Figure 1).

**Figure 1:**
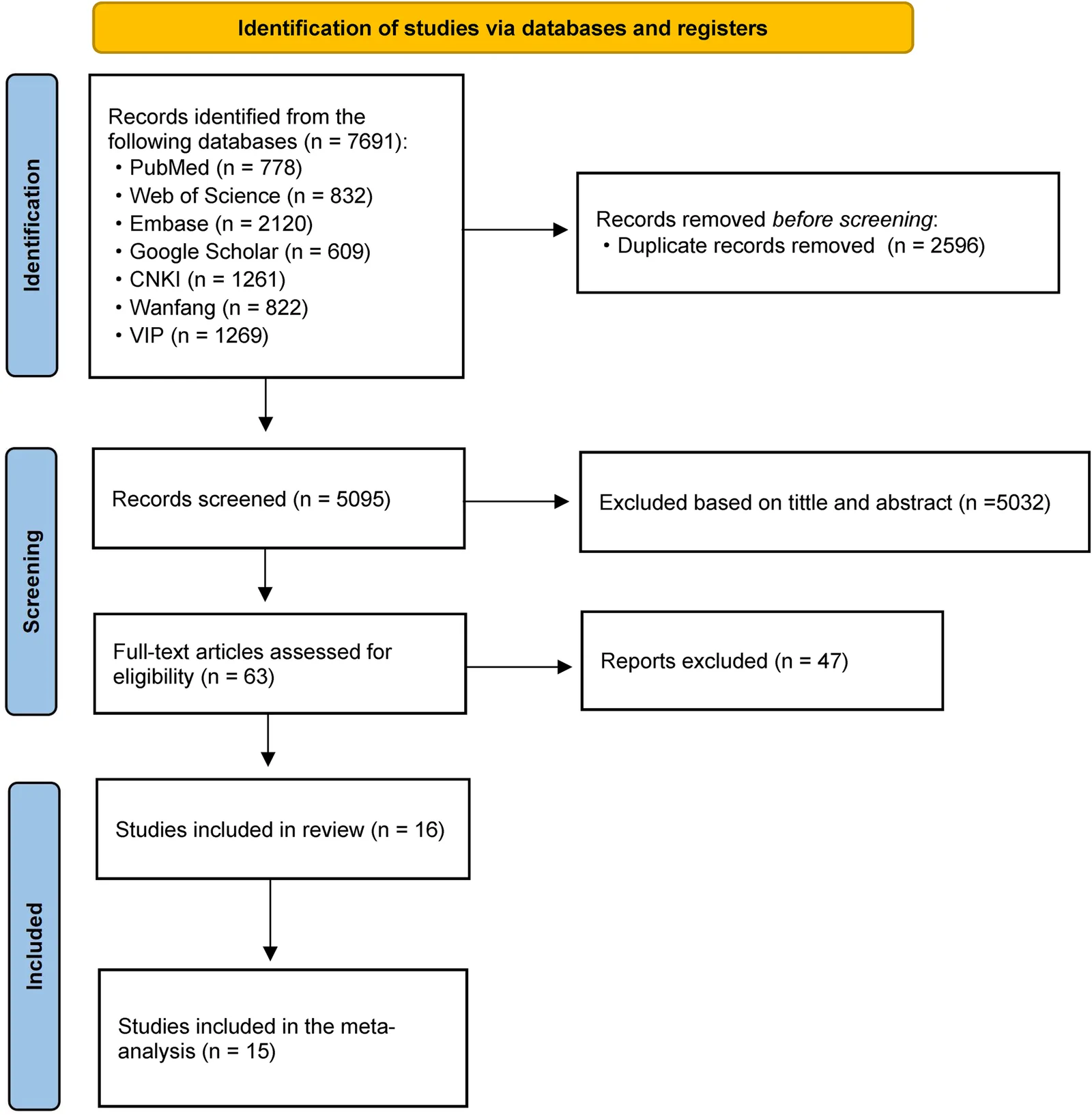
Preferred reporting items for systematic reviews and meta-analyses (PRISMA) flowchart of study selection.

The initial search yielded 7,691 records. After removing 2,596 duplicates, 5,095 titles and abstracts were screened for relevance. Of these, 63 full-text articles were assessed for eligibility, leading to the exclusion of 47 studies that did not meet the inclusion criteria. A total of 16 studies were included in the final systematic review (13–28), of which 15 were eligible for quantitative synthesis (13–23, 25–28).

### 3.2 Study characteristics and models validation

Table 1 summarizes the characteristics of the studies included in this systematic review and meta-analysis on TCM-WM integrated risk prediction models for CHD. A total of 16 studies conducted between 2008 and 2024 were analyzed, encompassing retrospective (*n* = 7), cross-sectional (*n* = 7), and prospective (*n* = 2) study designs. Most studies were carried out in China, with one study each from France and Korea. Participant populations varied widely, ranging from suspected coronary artery disease (CAD) patients to hypertensive and elderly individuals with ischemic symptoms, with age ranges typically spanning from young adulthood to late elderly (18–92 years). Data sources predominantly included university-affiliated hospitals, community healthcare facilities, and specialized cardiovascular centers. The proportion of CHD or CAD cases among total participants showed considerable variability, ranging from as low as 3.58% to as high as 80.54%, reflecting differences in study populations and diagnostic criteria.

**Table 1:** Characteristics of included studies on TCM-WM integrated CHD prediction models.

Author (Year)	Country	Study Design	Participants (Age Range)	Data Source (Institutes)	Outcome	Cases/Total (%)
Zhou Junge et al. (2015)	China	Retrospective	Suspected CAD with symptoms (24-88y)	Beijing Mentougou District Hospital	CAD	312/472 (66.10)
Zheng Chaonan et al. (2022)	China	Retrospective	First-diagnosis angina	First Affiliated Hospital of Henan University of Chinese Medicine	CHD	534/2459 (21.72)
Ding Yukun et al. (2023)	China	Prospective	AMI patients (≥18y)	Beijing Hai De Kang Jian IT Co.	CHD	73/653 (11.18)
Lin Zinuo et al. (2022)	China	Retrospective	Chest pain with first angiography	First Affiliated Hospital of Guangzhou University of Chinese Medicine	CHD	263/688 (38.23)
Zhou Zhihui et al. (2024)	China	Retrospective	No chronic diseases (25–85y)	Shanghai Shuguang Hospital & Community Elderly Center	CHD	300/406 (73.89)
Yang Yan et al. (2023)	China	Retrospective	Suspected CAD (≥18y)	Beijing Dongzhimen Hospital	CHD	567/877 (64.65)
Duan Mengyao et al. (2023)	China	Cross-sectional	General patients (18–85y)	First Affiliated Hospital of Hunan University of Chinese Medicine	CHD	315/526 (59.89)
Wang Chaojie et al. (2022)	China	Cross-sectional	Elderly with angina (≥60y)	Heilongjiang community facilities	CHD	274/738 (37.13)
Fang Rui et al. (2022)	China	Retrospective	Hypertensive elderly (60–85y)	Hangzhou TCM Hospital & Hunan University Hospitals	CHD	151/508 (29.72)
Jia Mengnan et al. (2022)	China	Retrospective	MI patients	First Affiliated Hospital of Henan University of TCM	CHD	255/2180 (11.70)
Yuanxi Xu et al. (2008)	China	Prospective	Suspected CAD (45–92y)	Shanghai Tongji Hospital	CAD	187/272 (68.75)
Xiao-Wu He et al. (2022)	China	Cross-sectional	Community adults (≥40y)	Taizhou rural clinics (Zhejiang)	CHD	251/7012 (3.58)
Mengyao Duan et al. (2024)	China	Cross-sectional	Hypertensive patients (18–85y)	Multiple Beijing & Hunan University Hospitals	CAD	166/410 (40.49)
Chang-Min Chung et al. (2015)	China	Cross-sectional	Elderly with ischemic symptoms (>65y)	Chiayi Chang Gung Memorial Hospital	CAD	298/370 (80.54)
Alexandre Vallée et al. (2019)	France	Cross-sectional	Adults with CV risk factors (≥18y)	Hôtel-Dieu University Hospital (Paris)	CHD	99/530 (18.68)
Young-Boum Lee et al. (2016)	Korea	Cross-sectional	CVD patients	Dongguk University Ilsan Hospital	CAD	80/246 (32.52)

TCM, Traditional Chinese Medicine; CAD, Coronary artery disease; CHD, Coronary heart disease; AMI, Acute myocardial infarction; MI, Myocardial infarction; CV, Cardiovascular; y, years.

Table 2 summarizes the methodological features of the included TCM-WM integrated CHD prediction models. Logistic regression was the most commonly applied method, alongside machine learning techniques such as decision trees (DT), random forests (RF), support vector machines (SVM), artificial neural networks (ANN), and extreme gradient boosting (XGBoost). Most studies selected predictors through both univariate and multivariate analyses and conducted internal validation; only a few performed external validation. Key TCM predictors included blood stasis, yin deficiency, phlegm, qi deficiency, and tongue image features. Pulse wave velocity and its derivatives also appeared recurrently as integrated predictors. Reported AUC values ranged from 0.64 to 0.97, but these study-level estimates should be interpreted in light of limited external validation and the frequent high or unclear risk of bias. Models were commonly presented using ROC curves, calibration plots, nomograms, and decision curve analysis.

**Table 2:** Methodological characteristics of TCM-WM integrated prediction models.

Author (Year)	Development Method	Variable Selection	Validation	Key TCM Predictors	Performance Metrics	Presentation Forms
Zhou Junge et al. (2015)	Logistic Regression	Uni + Multi	Internal	baPWV, ABI	OR = 8.44 (5.06–14.08)	-
Zheng Chaonan et al. (2022)	LR/DT/RF	Uni + Multi	External	Blood stasis, Yin deficiency	AUC=0.83, Acc=0.82	ROC, Calibration, Nomogram
Ding Yukun et al. (2023)	Logistic Regression	Uni + Multi	External	“Internal Collateral Wind” score	OR = 1.64 (1.36–1.99)	Nomogram, Calibration
Lin Zinuo et al. (2022)	LR/DT/RF	Uni + Multi	Internal	6 TCM syndromes^a^	AUC=0.82, Sens=64.2%	ROC, Nomogram, DCA
Zhou Zhihui et al. (2024)	Logistic Regression	Uni + Multi	Internal	Pulse wave velocity	OR = 3.57 (1.96–6.48)	ROC
Yang Yan et al. (2023)	LR/DT/RF/Group-sparse MLR	Uni + Multi	External	5 TCM syndromes, Tongue signs^b^	AUC=0.89, Acc=89.7%	ROC, Nomogram, Calibration, DCA
Duan Mengyao et al. (2023)	XGBoost/ANN/SVM	Multi	Internal	8 TCM syndromes, Tongue signs^b^	AUC=0.89	ROC, Nomogram, Calibration, DCA
Wang Chaojie et al. (2022)	Binary/Conditional LR	Multi	Internal	Yang deficiency, Phlegm-damp constitution	OR = 2.423 (1.098–4.453)	Forest plot, ROC
Fang Rui et al. (2022)	Binary/Conditional LR	Uni	Internal	Yin-yang imbalance, Qi deficiency & blood stasis	OR = 16.492 (8.312–32.724)	ROC
Jia Mengnan et al. (2022)	Binary/Conditional LR	Multi	External	Phlegm, Deficiency, Blood stasis	AUC=0.97 (0.95–0.99)	Nomogram, ROC, Calibration
Yuanxi Xu et al. (2008)	Logistic Regression	Uni + Multi	Internal	baPWV	OR = 1.00 (1.05–1.97)	Statistical tables
Xiao-Wu He et al. (2022)	Logistic Regression	Uni + Multi	Internal	Estimated pulse wave velocity	OR = 3.57 (1.96–6.48)	ROC
Mengyao Duan et al. (2024)	LR/DT/RF/XGBoost	Uni + Multi	Internal	Tongue image features	AUC=0.79	ROC
Chang-Min Chung et al. (2015)	LR/Linear Regression	Uni + Multi	Internal	baPWV	AUC=0.64, Sens=60.1%	ROC, Regression plot
Alexandre Vallée et al. (2019)	Decision Tree	Uni + Multi	External	Pulse wave velocity	AUC=0.87, Sens=62%	ROC
Young-Boum Lee et al. (2016)	Logistic Regression	Uni + Multi	Internal	Pulse wave velocity ratio	AUC=0.82	ROC

a 6 TCM syndromes included: Heart blood stasis, Qi stagnation & blood stasis, Phlegm turbidity obstruction, etc.

b Tongue signs included: Toothed tongue, Thick coating, Cracked tongue.

LR, logistic regression; DT, decision tree; RF, random forest; XGBoost, eXtreme Gradient Boosting; ANN, artificial neural network; SVM, support vector machine; MLR, multivariate logistic regression; Uni, univariate analysis; Multi, multivariable analysis; baPWV, Brachial-ankle Pulse Wave Velocity; ABI, ankle-brachial index; OR, odds ratio (with 95% Confidence Interval in parentheses); AUC, area under the receiver operating characteristic curve; Acc, Accuracy; Sens, Sensitivity; Spec, Specificity; ROC, receiver operating characteristic curve; DCA, decision curve analysis.

### 3.3 Quality assessment

Table 3 presents the risk of bias and applicability assessments based on the PROBAST tool. Among the 16 studies, 11 were development-only studies (type A) and 5 included both development and validation (type B). Four studies were judged to have an overall low risk of bias, while twelve studies were rated as high or unclear risk. The analysis domain exhibited the highest frequency of high or unclear risk of bias. Regarding applicability, 13 studies showed low concerns across all domains, whereas 3 studies exhibited high concerns, primarily related to participant selection.

**Table 3:** Risk of bias and applicability assessment using PROBAST tool.

Author (Year)	Study Type	Risk of Bias (ROB) Domain				Applicability Concern Domain			Overall	
		Participants	Predictors	Outcome	Analysis	Participants	Predictors	Outcome	ROB	Applicability
Zhou Junge et al. (2015)	A	+	+	+	?	+	+	+	?	+
Zheng Chaonan et al. (2022)	B	+	+	+	+	+	+	+	+	+
Ding Yukun et al. (2023)	B	-	+	+	-	+	+	+	-	+
Lin Zinuo et al. (2022)	A	+	+	+	+	+	+	+	+	+
Zhou Zhihui et al. (2024)	A	+	-	+	?	+	+	+	-	+
Yang Yan et al. (2023)	B	+	+	+	+	+	+	+	+	+
Duan Mengyao et al. (2023)	A	+	-	+	?	+	+	+	-	+
Wang Chaojie et al. (2022)	A	+	-	+	-	-	+	+	-	-
Fang Rui et al. (2022)	A	+	+	+	?	+	+	+	?	+
Jia Mengnan et al. (2022)	B	+	+	-	+	-	-	+	-	-
Yuanxi Xu et al. (2008)	A	+	+	+	-	+	+	+	-	+
Xiao-Wu He et al. (2022)	A	-	+	+	-	-	+	+	-	-
Mengyao Duan et al. (2024)	A	+	+	+	-	+	+	+	-	+
Chang-Min Chung et al. (2015)	A	+	+	-	?	+	+	+	-	+
Alexandre Vallée et al. (2019)	B	+	+	+	+	+	+	+	+	+
Young-Boum Lee et al. (2016)	A	?	+	+	-	+	+	+	-	+

PROBAST: Prediction model Risk Of Bias Assessment Tool.

A: Development only; B: Development with validation.

ROB: Risk of bias.

+: Low risk/concern; -: High risk/concern; ?: Unclear risk/concern.

### 3.4 Meta-analysis for sensitivity, specificity and area under the curve of the included models

The meta-analysis synthesized diagnostic-performance data from 12 studies. The pooled sensitivity was 0.79 (95% CI: 0.62–0.89), with extreme heterogeneity (I² = 98.17%), and the pooled specificity was 0.87 (95% CI: 0.76–0.94), also with extreme heterogeneity (I² = 99.39%) (Figure 2).

**Figure 2:**
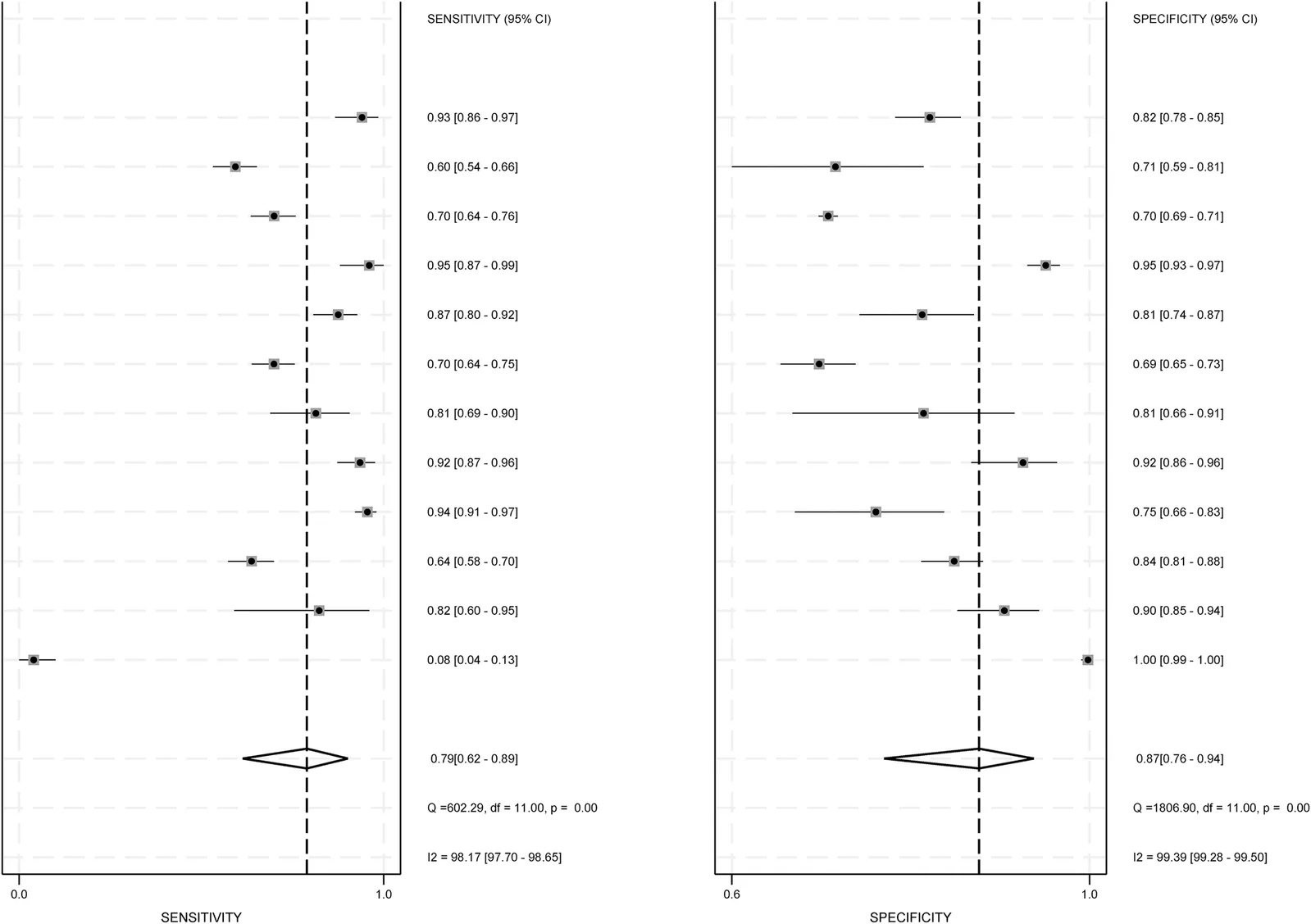
Forest plot of the random model meta-analysis of pooled sensitivity and specificity for the 12 studies.

The summary receiver operating characteristic (SROC) analysis of the model demonstrated powerful diagnostic accuracy, with an AUC of 0.90 (95% CI: 0.88–0.93), indicating excellent discriminatory performance (Figure 3).

**Figure 3:**
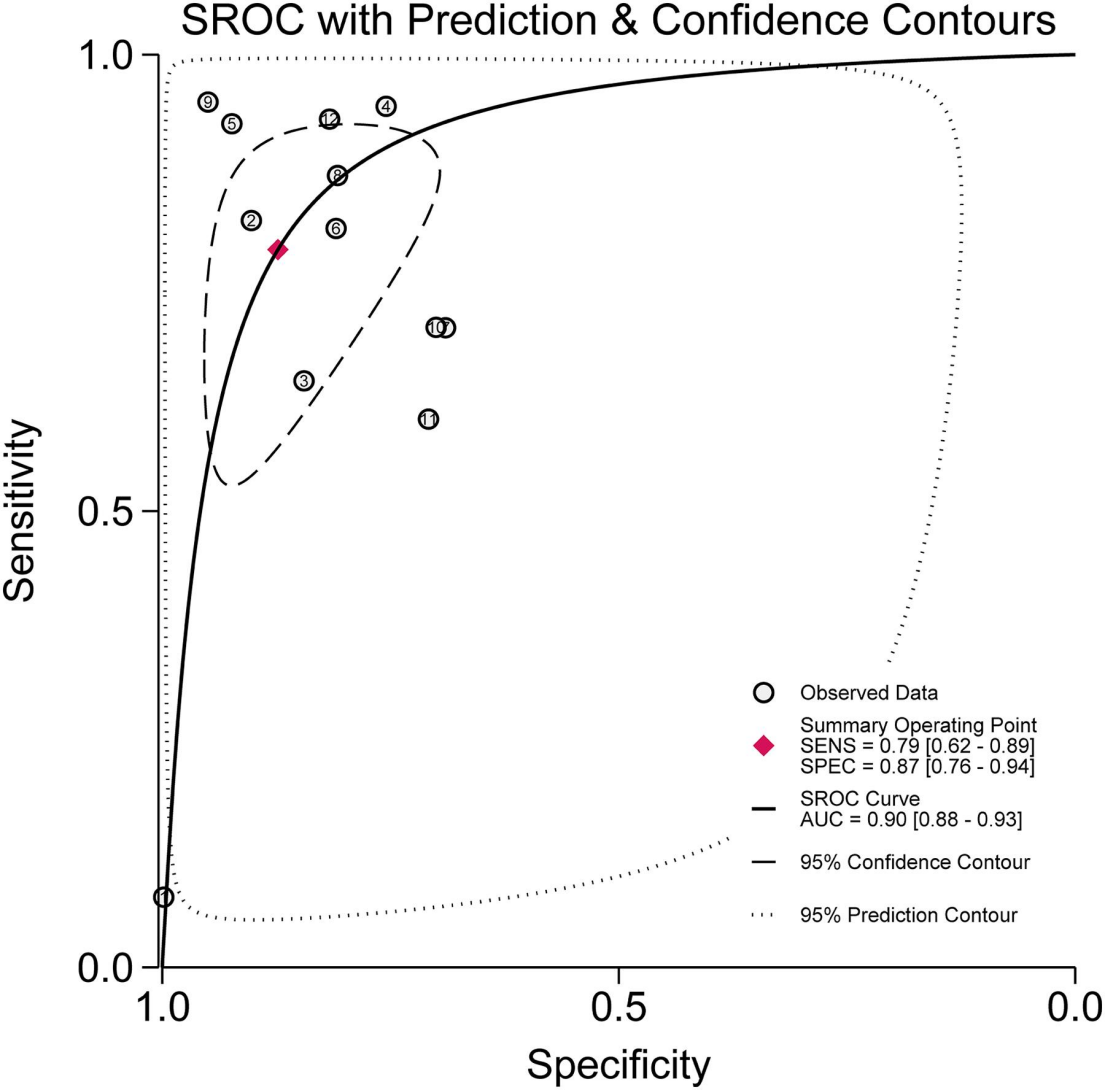
Receiver operating characteristics curves of the random model meta-analysis of pooled area under the curve for the 12 studies.

The Deeks’ funnel plot asymmetry test assessing sensitivity, specificity and AUC demonstrated borderline non-significant asymmetry (*p* = 0.08), with visual inspection revealing minor funnel plot asymmetry (Supplementary Figure 1).

### 3.5 Meta-analysis for odds ratio of the included models

Across 7 studies, the OR meta-analysis showed extreme heterogeneity (I² = 95.6%, *p* < 0.001). The random-effects pooled OR was 1.80 (95% CI: 1.40–2.30) (Figure 4); however, because the study effects were highly inconsistent, this value should be viewed as an average association rather than a precise or broadly generalizable effect estimate.

**Figure 4:**
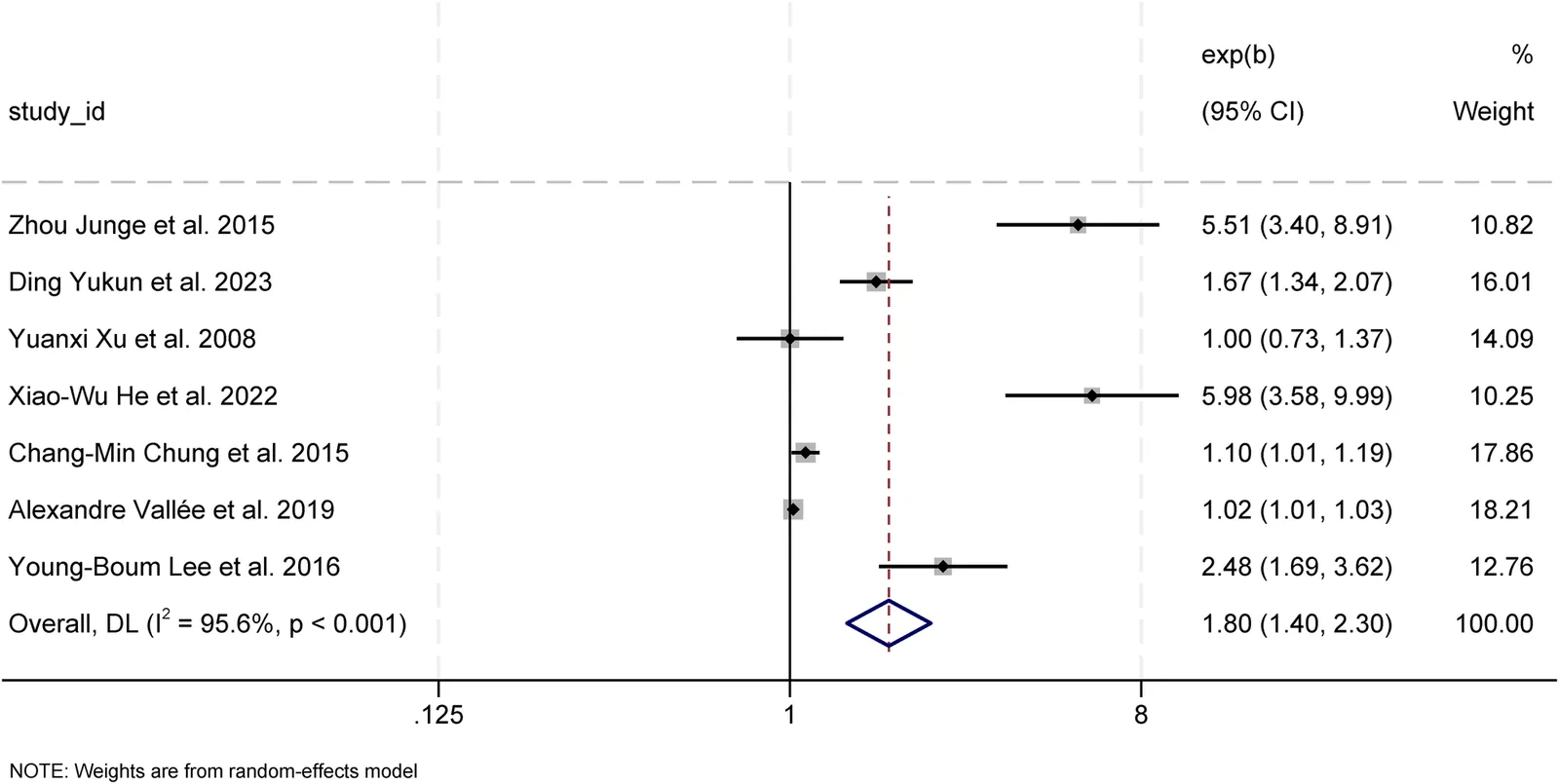
Forest plot of the random model meta-analysis of pooled odds ratio for the 7 studies.

Notably, two studies exhibited particularly strong effects: Zhou Junge et al. (OR = 5.51, 95% CI: 3.40–8.91) and Xiao-Wu He et al. (OR = 5.98, 95% CI: 3.58–9.99). However, three studies showed non-significant or marginal effects, with OR point estimates close to null (Yuanxi Xu et al.: OR = 1.00, 95% CI: 0.73–1.37; Alexandre Vallée et al.: OR = 1.02, 95% CI: 1.01–1.03).

The Deeks’ funnel plot asymmetry test assessing OR demonstrated no statistically significant asymmetry (*p* = 0.37), with visual inspection revealing minor funnel plot asymmetry (Supplementary Figure 2).

### 3.6 Meta-regression

We performed univariable meta-regression and subgroup analyses based on two prespecified TCM-related characteristics: 1) the level of standardization in TCM diagnosis and treatment (high level vs. low level), and 2) the number of TCM predictors included in the model (multivariate vs. univariate).

As shown in Figure 5, studies employing standardized TCM diagnostic methodologies (*n* = 7) achieved a pooled sensitivity of 0.75 (95% CI: 0.55–0.94) and a pooled specificity of 0.89 (95% CI: 0.80–0.99). In comparison, studies without standardized TCM approaches (*n* = 5) yielded a slightly higher pooled sensitivity of 0.83 (95% CI: 0.66–1.00) and a marginally lower specificity of 0.84 (95% CI: 0.69–0.99). Statistical analysis indicated no significant differences in sensitivity (*P* = 0.40) or specificity (*P* = 0.92) between these two groups. Meta-regression further confirmed that TCM standardization was not a significant source of heterogeneity (*P* = 0.80).

**Figure 5:**
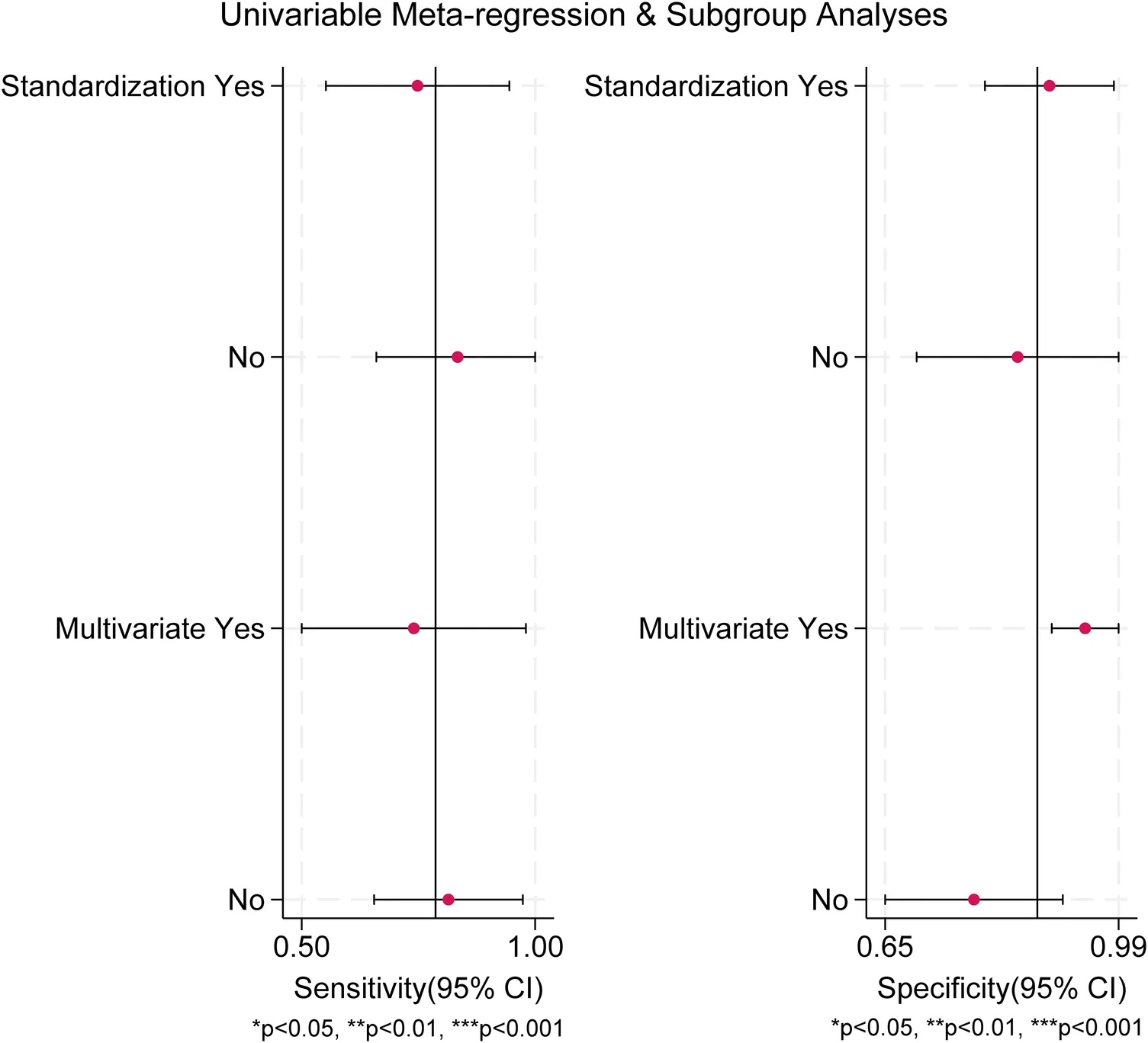
Univariable meta-regression and subgroup analyses of Traditional Chinese medicine-related characteristics.

Regarding the number of TCM predictors, studies incorporating multivariate TCM factors (*n* = 5) had a pooled sensitivity of 0.74 (95% CI: 0.50–0.98) and specificity of 0.94 (95% CI: 0.89–0.99), whereas studies using univariate TCM predictors (*n* = 7) had a pooled sensitivity of 0.81 (95% CI: 0.65–0.97) and specificity of 0.78 (95% CI: 0.65–0.91). The between-group comparisons were not statistically significant for sensitivity (*P* = 0.47) or specificity (*P* = 0.43). Meta-regression for specificity reached the conventional significance threshold (*P* = 0.05), suggesting a possible source of heterogeneity.

### 3.7 Subgroup analyses

We conducted additional subgroup analyses based on all TCM and WM predictors included in the studies, and pooled their corresponding ORs.

For TCM-related factors, the results were as follows: pulse wave velocity (OR = 1.25, 95% CI: 1.06–1.46, I² = 91.2%, *p* < 0.001), TCM pattern (OR = 1.54, 95% CI: 0.9–2.50, I² = 85.6%, *p* < 0.001), TCM pattern unit (OR = 26.4, 95% CI: 2.41–289.10, I² = 92%, *p* < 0.001), qi deficiency syndrome (OR = 0.6, 95% CI: 0.04–8.67, I² = 89.9%, *p* = 0.002), blood stasis syndrome (OR = 0.6, 95% CI: 0.15–2.37, I² = 88.5%, *p* < 0.001), and yang deficiency syndrome (OR = 2.36, 95% CI: 0.69–8.05, I² = 74.5%, *p* = 0.048) (Supplementary Figures 3−8).

For WM-related factors, the results included: hypertension history (OR = 1.61, 95% CI: 1–2.58, I² = 81.4%, *p* < 0.001), diabetes history (OR = 2.58, 95% CI: 2–3.33, I² = 0%, *p* = 0.896), smoking history (OR = 1.53, 95% CI: 0.14–17.04, I² = 97.5%, *p* < 0.001), hyperlipidemia (OR = 2.15, 95% CI: 0.86–5.35, I² = 84.5%, *p* = 0.002), clinical symptoms of coronary heart disease (OR = 11.33, 95% CI: 5.91–21.74, I² = 57.6%, *p* = 0.125), age (OR = 1.05, 95% CI: 1.01–1.10, I² = 84.9%, *p* < 0.001), and sex (OR = 1.3, 95% CI: 0.52–3.24, I² = 91.4%, *p* < 0.001) (Supplementary Figures 9−15).

### 3.8 Leave-one-out sensitivity analyses

For the diagnostic-performance analysis, sequential exclusion of individual studies did not materially alter the pooled sensitivity, specificity, or AUC estimates (Supplementary Table S2).

Compared with the original pooled estimates (sensitivity: 0.79, 95% CI: 0.62–0.89; specificity: 0.87, 95% CI: 0.76–0.94; AUC: 0.90, 95% CI: 0.88–0.93), pooled sensitivity after removing individual studies ranged from 0.76 to 0.84, specificity ranged from 0.83 to 0.88, and AUC ranged from 0.89 to 0.92. Exclusion of Zheng Chaonan et al. (2022) produced the highest pooled sensitivity, whereas exclusion of Zhou Zhihui et al. (2024) or Jia Mengnan et al. (2022) produced the lowest. The lowest AUC followed exclusion of Yang Yan et al. (2023) or Jia Mengnan et al. (2022), and the highest followed exclusion of Chang-Min Chung et al. (2015).

For the pooled OR analysis, leave-one-out sensitivity analysis likewise showed that no single study fully determined the pooled association (Supplementary Table S3), but the analysis did not resolve the extreme heterogeneity.

The original pooled OR was 1.80 (95% CI: 1.40–2.30), and sequential exclusion of individual studies yielded pooled ORs from 1.52 to 2.21. The lowest pooled estimate followed exclusion of Zhou Junge et al. (2015) or Xiao-Wu He et al. (2022), whereas exclusion of Alexandre Vallée et al. (2019) produced the highest estimate. All leave-one-out point estimates remained greater than 1. Nevertheless, their persistence does not establish a uniform effect because between-study heterogeneity remained extreme.

## 4 Discussion

The sensitivity of CAD risk scores is closely linked to the heterogeneity of individual disease presentation, and predictive models have played an increasingly important role in non-invasive diagnostics (29). In recent years, AI-driven risk prediction models have been widely introduced into the detection of CAD, with ongoing advances shifting from traditional statistical approaches toward machine learning (ML) and even deep learning (DL) methodologies (30). In the emerging field of intelligent medicine, the integration of TCM and WM represents a promising direction for cardiovascular care and may contribute to enhancing the accuracy of prediction models (31).

Previous studies have reported that WM-based CAD risk prediction models achieved an AUC of 0.81 for predicting 10-year incident CAD risk (32). Another meta-analysis using boosting algorithms reported a higher pooled AUC of 0.88 (95% CI: 0.84–0.91) (33). In addition, a comparative study evaluating 28 CAD risk prediction models concluded that no single model can yet be recommended for routine CAD risk prediction (34).

This systematic review included 16 studies of TCM-WM integrated risk prediction models for CHD. The meta-analysis yielded pooled estimates of 0.79 for sensitivity, 0.87 for specificity, and 0.90 for AUC, and an OR of 1.80 across the 7 studies contributing to that analysis. These numerical averages appear favorable, but they do not demonstrate consistently high or reliable performance: heterogeneity was extreme for sensitivity (I² = 98.17%), specificity (I² = 99.39%), and OR (I² = 95.6%). The pooled estimates therefore have low certainty and may not reflect performance in any particular population, institution, or diagnostic pathway. The findings support further investigation of TCM variables, but they do not establish an independent incremental benefit over WM-only models or justify clinical implementation.

Importantly, these integrated models should not be viewed as alternatives to established diagnostic procedures such as coronary angiography. Their potential clinical role is more appropriately positioned as pretest risk-stratification or decision-support tools that combine routinely available WM variables with standardized, non-invasive TCM-related information before definitive diagnostic testing (35, 36). In this context, the potential value of TCM-related predictors would lie in providing complementary information for risk assessment rather than replacing objective WM diagnostic criteria.

Subgroup analyses and meta-regression explored whether TCM standardization and the number of TCM predictor categories accounted for heterogeneity. Standardized TCM methodology was not associated with statistically significant differences in sensitivity or specificity. Models with multiple TCM predictor categories had a higher subgroup-specificity estimate than models with one category, but the direct between-group comparison was not statistically significant (*P* = 0.43), and the meta-regression result for specificity was borderline (*P* = 0.05). Given the small number of studies, multiple comparisons, non-randomized study-level contrasts, and residual heterogeneity, these results should be treated as exploratory. They suggest hypotheses about how TCM information might be structured, but they do not demonstrate an additive effect or explain most of the observed inconsistency.

Additional subgroup analyses pooled ORs for six TCM and seven WM factors. Point estimates for qi deficiency and blood stasis syndromes were below 1, but their wide confidence intervals included the null. These factors therefore cannot be classified as protective on the present evidence. Most other factor-specific estimates were also imprecise and highly heterogeneous. The descriptive mean of the TCM-factor ORs (5.46) exceeded that of the WM-factor ORs (3.08), but this unweighted comparison does not account for precision, outcome definition, scaling, or the number of contributing studies and should not be interpreted as evidence that TCM factors have stronger predictive ability. Only diabetes history and clinical symptoms of CHD lacked statistically significant heterogeneity. The remaining analyses generally showed substantial or extreme inconsistency. These findings underscore uncertainty arising from differing populations, definitions, measurement procedures, and modeling strategies rather than establishing stable factor effects.

A more informative approach to TCM-WM integration may be to examine cross-domain relationships between TCM syndrome elements and established cardiovascular risk factors rather than considering the two domains separately. For example, future studies could investigate whether specific syndrome elements are associated with lipid profiles, blood pressure, glycemic indices, or other objective cardiovascular markers (37–39). Such analyses may help clarify the biological correlates of TCM-related features and determine whether they provide complementary information beyond conventional WM predictors (37, 39). However, the studies included in this review did not provide sufficiently consistent paired data to quantitatively evaluate these relationships. Prospective studies integrating standardized TCM phenotyping with objective biomedical measurements are therefore warranted.

The classification of PWV warrants particular consideration. Although PWV is widely recognized in conventional cardiovascular medicine as an indicator of arterial stiffness, pulse-wave acquisition and quantitative pulse analysis have also been employed in the modernization and objectification of pulse-related assessment in TCM (7, 40). In this study, PWV and related pulse-wave parameters were therefore classified as TCM-related when they represented quantitative extensions of pulse-related assessment within the integrative diagnostic framework. This classification should not be interpreted as indicating that PWV is specific or exclusive to TCM. Rather, it reflects the methodological overlap that can arise when traditional pulse diagnostic information is translated into quantitative physiological measurements (7, 40, 41). Such overlap also highlights the need for future integrative prediction studies to clearly specify the theoretical basis and intended diagnostic role of cross-domain predictors.

In terms of study characteristics, the majority of included studies were conducted in China (14/16, 87.5%) and within hospital settings (12/16, 75%), which substantially limits the generalizability of findings to other geographic regions and healthcare environments. The study designs spanned cross-sectional (7/16, 43.75%), retrospective (7/16, 43.75%), and prospective (2/16, 12.5%) observational studies, with a relatively low proportion of high-evidence-level prospective investigations. Considerable heterogeneity in patient characteristics was noted, primarily due to inconsistent diagnostic criteria across studies. While coronary angiography is widely accepted as the gold standard for CHD diagnosis, some studies relied on clinical symptoms or electrocardiographic findings, potentially compromising diagnostic accuracy. The integration of AI has improved diagnostic precision in some cases (42). Nonetheless, the lack of standardized diagnostic references and incomplete reporting of clinical assessment procedures remains a major source of heterogeneity and limits the comparability of findings.

Regarding model characteristics, logistic regression was the most commonly used method (13/16, 81.25%), with the majority of studies (15/16, 93.75%) employing either conventional statistical techniques or machine learning approaches. Notably, nine studies adopted multiple algorithms to explore differences in predictive performance, seeking optimal modeling strategies. Logistic regression, a classical statistical method, offers certain advantages in integrated TCM-WM prediction models due to its interpretability, compatibility with small sample sizes, and ability to incorporate diverse variable types (43). Traditional statistical and ML methods were considered more appropriate in this context, as DL models often require large datasets, pose interpretability challenges [necessitating tools such as SHapley Additive exPlanations (SHAP)], and carry a higher risk of overfitting (44).

With respect to variable selection and model validation, 12 studies applied a combination of univariate and multivariate methods, and several compared multiple algorithms. Because these comparisons differed in predictors, samples, and validation procedures, the review cannot determine that one modeling approach produced stronger or more generalizable effects. Only 5 of 16 studies (31.25%) conducted external validation, raising substantial concerns about overfitting and optimism in reported performance (45). This concern is amplified by the geographic and institutional concentration of the evidence. Future models require multicenter prospective development, adequately sized samples, transparent reporting, and independent external validation before their clinical applicability can be judged (45, 46).

The available evidence raises the hypothesis that systematically measured TCM variables, including tongue appearance, pulse characteristics, syndrome differentiation, and elemental patterns, may contribute useful information to integrated models. However, the present meta-analysis did not directly quantify incremental performance against otherwise identical WM-only models. The exploratory meta-regression concerning multiple TCM predictors was based on few studies and did not establish a statistically robust improvement in specificity. Any additive value of a multidimensional TCM framework therefore requires prospective head-to-head evaluation with calibration, discrimination, clinical utility, and external validation reported (46, 47).

This systematic review is among the first to focus specifically on TCM-WM integrated approaches for CHD diagnosis. Multimodal integration of physiological, clinical, traditional diagnostic, and AI-derived data has a plausible rationale in complex cardiovascular disease (48, 49), and non-invasive TCM indicators could be feasible to collect where advanced imaging is limited (49). Nevertheless, the present evidence does not show that adding TCM indicators improves accuracy, outcomes, or resource use compared with WM-only models. These potential advantages remain hypotheses for prospective comparative studies.

From a clinical perspective, TCM-WM integrated models could be translated into practical risk-assessment tools by combining standardized TCM-related features with routinely available WM clinical variables. These models could be incorporated into electronic health records, web-based risk calculators, or clinical decision-support systems to generate an individualized probability or risk category for CHD (36, 47). Such information could support pretest risk stratification and help clinicians determine whether further diagnostic evaluation is warranted, rather than replacing established diagnostic procedures (35, 36). Before bedside implementation, however, these tools require prospective external validation, calibration assessment, evaluation of clinical utility, and standardized acquisition of TCM-related information (45, 46).

## 5 Limitations and future directions

This study has several important limitations that materially reduce certainty in the pooled estimates. First, heterogeneity was extreme for the main outcomes (sensitivity I² = 98.17%, specificity I² = 99.39%, and OR I² = 95.6%). A random-effects model summarizes this variability but does not remove it, and leave-one-out stability only shows that no single study drove the averages. The pooled values therefore should not be interpreted as precise estimates of expected performance in a new setting. The prespecified subgroup analyses and meta-regression did not adequately explain the inconsistency and were underpowered because few studies contributed to each comparison. Second, 12 of 16 studies had high or unclear overall risk of bias, particularly in the analysis domain, and external validation was reported in only 5 studies. These limitations increase the likelihood of overfitting and optimistic performance estimates. Third, most studies were conducted in China and in hospital settings, restricting applicability to other regions and primary care. Study populations, CHD definitions, reference standards, TCM diagnostic criteria, predictor measurement, and modeling methods varied substantially; some studies used clinical symptoms or electrocardiographic findings instead of coronary angiography, further reducing comparability. Fourth, small samples and incomplete reporting limited assessment of calibration and clinical utility. Finally, although Deeks’ tests did not detect statistically significant funnel-plot asymmetry, the small number of studies provides limited power to exclude publication bias.

Future research should prioritize multicenter, prospective studies with larger and more diverse populations and prespecified external validation in independent cohorts. Harmonized definitions and measurement protocols are needed for CHD outcomes, reference standards, TCM syndromes, tongue and pulse features, and predictor timing. Studies should compare integrated models directly with appropriate WM-only benchmarks and report incremental discrimination, calibration, decision-curve analysis, and clinically relevant consequences. Interpretable machine-learning methods may be evaluated when sample size and validation procedures are adequate, but added algorithmic complexity should not substitute for representative data or transparent methods. Full reporting according to TRIPOD and structured risk-of-bias assessment using PROBAST are essential. With sufficient studies, future meta-analyses should also report prediction intervals and investigate heterogeneity using adequately powered, prespecified analyses.

## 6 Conclusions

This systematic review and meta-analysis found favorable average estimates for TCM-WM integrated CHD prediction models (sensitivity 0.79, specificity 0.87, and AUC 0.90). However, extreme heterogeneity in sensitivity and specificity, frequent high or unclear risk of bias, and limited external validation substantially undermine confidence in these pooled values. They should not be interpreted as reliable performance estimates for routine clinical use or as evidence that integrated models outperform WM-only alternatives.

The OR analyses were likewise extremely heterogeneous, and exploratory factor-level and meta-regression findings do not establish that TCM predictors are protective, have greater predictive ability than WM factors, or significantly improve model performance. At present, the evidence supports the potential value of studying standardized TCM information, but the magnitude and generalizability of any incremental benefit remain uncertain.

Before clinical implementation, integrated models require transparent development, direct comparison with WM-only benchmarks, standardized TCM data collection, and rigorous multicenter external validation. The present findings should be viewed as hypothesis-generating evidence that defines a research agenda, not as confirmation of dependable clinical performance.

From a clinical perspective, future research should determine whether externally validated TCM-WM models can support pretest risk stratification and clinical decision-making, while establishing whether TCM-related information provides meaningful complementary value beyond conventional WM predictors.

## Data Availability

The original contributions presented in the study are included in the article/Supplementary Material, further inquiries can be directed to the corresponding authors.
