# Supplementary material for: Traditional Chinese and Western medicine-integrated risk prediction models for coronary heart disease: a systematic review and meta-analysis
Source: Front Cardiovasc Med. 2026 Sep 25;13:1921708. doi: 10.3389/fcvm.2026.1921708 (PMC13649438; doi:10.3389/fcvm.2026.1921708)
Supplement: Supplementary file 1 [file Datasheet1.docx]

Supplementary Table 1. The detailed search strategies of the study.

| Database | Search Strategy | Results |
| --- | --- | --- |
| PubMed | (("Coronary Artery Disease"[MeSH] OR "Myocardial Ischemia"[MeSH] OR "Angina Pectoris"[MeSH] OR "Myocardial Infarction"[MeSH] OR "coronary heart disease" OR "CAD" OR "myocardial ischemia" OR "angina" OR "heart attack" OR "coronary disease" OR "coronary artery disease diagnosis" OR "coronary artery disease treatment" OR "coronary artery disease prevention" OR "coronary artery disease risk factors" OR "coronary artery disease pathogenesis" OR "coronary artery disease complications")) AND (("Medicine, Chinese Traditional"[MeSH] OR "Acupuncture Therapy"[MeSH] OR "Drugs, Chinese Herbal"[MeSH] OR "Integrative Medicine"[MeSH] OR "Traditional Chinese Medicine" OR "TCM" OR "Chinese herbal medicine" OR "acupuncture" OR "syndrome differentiation" OR "integrated traditional Chinese and Western medicine" OR "combination of Chinese and Western medicine" OR "Chinese medicine syndrome" OR "TCM treatment" OR "TCM diagnosis" OR "TCM theory" OR "TCM practice" OR "syndrome element" OR "syndrome pattern" OR "tongue" OR "tongue diagnosis" OR "pulse" OR "pulse diagnosis" OR "constitution" OR "TCM constitution")) AND (("Models, Statistical"[MeSH] OR "Risk Assessment"[MeSH] OR "Machine Learning"[MeSH] OR "Algorithms"[MeSH] OR "predictive model" OR "risk assessment model" OR "statistical model" OR "clinical prediction model" OR "model construction" OR "model validation" OR "model evaluation" OR "model optimization" OR "model application" OR "machine learning model" OR "artificial intelligence model" OR "decision tree model" OR "logistic regression model")) | 778 |
| Web of Science | (("traditional Chinese medicine" OR "TCM" OR "Chinese herbal medicine" OR "acupuncture" OR "syndrome differentiation" OR "integrated traditional Chinese and Western medicine" OR "combination of Chinese and Western medicine" OR "Chinese medicine syndrome" OR "TCM rehabilitation" OR "TCM efficacy evaluation" OR "syndrome" OR "symptom" OR "pattern differentiation" OR "constitution" OR "tongue" OR "pulse" OR "sign" OR "TCM syndrome" OR "syndrome elements" OR "pattern type") AND ("coronary heart disease" OR "CAD" OR "myocardial ischemia" OR "angina" OR "heart attack" OR "coronary artery disease" OR "myocardial infarction" OR "chest pain" OR "coronary disease" OR "coronary artery disease risk stratification" OR "coronary artery disease prognosis" OR "coronary artery disease epidemiology") AND ("predictive model" OR "risk assessment model" OR "clinical prediction model" OR "machine learning model" OR "statistical model" OR "model validation" OR "model evaluation" OR "model optimization" OR "model application" OR "random forest" OR "neural network" OR "support vector machine" OR "ROC curve" OR "AUC value" OR "model calibration")) | 832 |
| Embase | #1 'coronary heart disease':ti,ab  #2 'CAD':ti,ab  #3 'myocardial ischemia':ti,ab  #4 'angina':ti,ab  #5 'heart attack':ti,ab  #6 'coronary disease':ti,ab  #7 'coronary artery disease diagnosis':ti,ab  #8 'coronary artery disease treatment':ti,ab  #9 'coronary artery disease prevention':ti,ab  #10 'coronary artery disease'/exp  #11 'myocardial ischemia'/exp  #12 'angina pectoris'/exp  #13 'myocardial infarction'/exp  #14 #1 OR #2 OR #3 OR #4 OR #5 OR #6 OR #7 OR #8 OR #9  OR #10 OR #11 OR #12 OR #13  #15 'Traditional Chinese Medicine':ti,ab  #16 'Chinese medicine':ti,ab  #17 'TCM':ti,ab  #18 'syndrome differentiation':ti,ab  #19 'integrated traditional Chinese and Western medicine':ti,ab  #20 'combination of Chinese and Western medicine':ti,ab  #21 'Chinese medicine syndrome':ti,ab  #22 'syndrome element':ti,ab  #23 'syndrome pattern':ti,ab  #24 'sign':ti,ab  #25 'tongue':ti,ab  #26 'tongue diagnosis':ti,ab  #27 'pulse':ti,ab  #28 'pulse diagnosis':ti,ab  #29 'constitution':ti,ab  #30 'TCM constitution':ti,ab  #31 'Traditional Chinese Medicine'/exp  #32 'Chinese medicine'/exp  #33 'integrative medicine'/exp  #34 #15 OR #16 OR #17 OR #18 OR #19 OR #20 OR #21 OR #22 OR #23 OR #24 OR #25 OR #26 OR #27 OR #28 OR #29 OR #30 OR #31 OR #32 OR #33  #35 'predictive model':ti,ab  #36 'risk assessment model':ti,ab  #37 'clinical prediction model':ti,ab  #38 'machine learning model':ti,ab  #39 'statistical model':ti,ab  #40 'model validation':ti,ab  #41 'model evaluation':ti,ab  #42 'model optimization':ti,ab  #43 'model application':ti,ab  #44 'predictive model'/exp  #45 'risk assessment'/exp  #46 'machine learning'/exp  #47 'statistical model'/exp  #48 #35 OR #36 OR #37 OR #38 OR #39 OR #40 OR #41 OR #42 OR #43 OR #44 OR #45 OR #46 OR #47  #49 #14 AND #34 AND #48 | 755 |
| Google Scholar | "Coronary Artery Disease" AND "Chinese medicine" AND ("predictive model" OR "risk assessment model" OR "statistical model" OR "clinical prediction model" ) | 609 |
| VIP | (M='冠心病' OR M='冠状动脉粥样硬化性心脏病' OR M='心肌缺血' OR M='心绞痛' OR M='心肌梗死' OR M='冠心病诊断' OR M='冠心病治疗' OR M='冠心病预防' OR M='冠心病危险因素' OR M='冠心病病机' OR M='冠心病并发症') AND (M='中医' OR M='中医临床' OR M='中医诊断' OR M='中医治疗' OR M='辨证论治' OR M='中西医结合' OR M='中医药治疗' OR M='中西医结合疗法' OR M='中西医结合医学' OR M='中西医结合临床' OR M='证型' OR M='证候' OR M='中西医结合治疗' OR M='中西医结合研究' OR M='中西医结合理论' OR M='中西医结合实践' OR M='中西医结合优势') AND (M='预测模型' OR M='风险评估模型' OR M='统计模型' OR M='临床预测模型' OR M='预测模型构建' OR M='预测模型验证' OR M='预测模型评估' OR M='预测模型优化' OR M='预测模型应用') | 1269 |
| CNKI | (TKA='冠心病' OR TKA='冠状动脉粥样硬化性心脏病' OR TKA='心肌缺血' OR TKA='心绞痛' OR TKA='心肌梗死' OR TKA='冠心病诊断' OR TKA='冠心病治疗' OR TKA='冠心病预防' OR TKA='冠心病危险因素' OR TKA='冠心病病机' OR TKA='冠心病并发症' OR TKA='胸痹' OR TKA='心痛') AND (TKA='中医' OR TKA='中医临床' OR TKA='中医诊断' OR TKA='中医治疗' OR TKA='辨证论治' OR TKA='中西医结合' OR TKA='中西医结合疗法' OR TKA='中西医结合医学' OR TKA='中西医结合临床' OR TKA='中医药治疗' OR TKA='证型' OR TKA='证候' OR TKA='证素' OR TKA='中西医结合治疗' OR TKA='中西医结合研究' OR TKA='中西医结合理论' OR TKA='中西医结合实践' OR TKA='中西医结合优势' OR TKA='中医思维' OR TKA='病证结合' OR TKA='症状' OR TKA='体征' OR TKA='舌' OR TKA='脉' OR TKA='体质') AND (TKA='预测模型' OR TKA='风险评估模型' OR TKA='统计模型' OR TKA='临床预测模型' OR TKA='预测模型构建' OR TKA='预测模型验证' OR TKA='预测模型评估' OR TKA='预测模型优化' OR TKA='预测模型应用' OR TKA='机器学习模型' OR TKA='人工智能模型' OR TKA='决策树模型' OR TKA='Logistic回归模型') | 1261 |
| Wanfang | (摘要:('冠心病' OR '冠状动脉粥样硬化性心脏病' OR '心肌缺血' OR '心绞痛' OR '心肌梗死' OR '冠心病诊断' OR '冠心病治疗' OR '冠心病预防' OR '冠心病危险因素' OR '冠心病病机' OR '冠心病并发症' OR '胸痹' OR '心痛')  OR 主题词:('冠心病' OR '冠状动脉粥样硬化性心脏病' OR '心肌缺血' OR '心绞痛' OR '心肌梗死' OR '胸痹' OR '心痛') OR 关键词:('冠心病' OR '冠状动脉粥样硬化性心脏病' OR '心肌缺血' OR '心绞痛' OR '心肌梗死' OR '冠心病诊断' OR '冠心病治疗' OR '冠心病预防' OR '冠心病危险因素' OR '冠心病病机' OR '冠心病并发症' OR '胸痹' OR '心痛')) AND (摘要:('中医' OR '中医临床' OR '中医诊断' OR '中医治疗' OR '辨证论治' OR '中西医结合' OR '中医药治疗' OR '中西医结合疗法' OR '中西医结合医学' OR '中西医结合临床' OR '证型' OR '证候' OR '证素' OR '症状' OR '体征' OR '舌' OR '脉' OR '体质' OR '中西医结合治疗' OR '中西医结合研究' OR '中西医结合理论' OR '中西医结合实践' OR '中西医结合优势' OR '中医思维' OR '病证结合') AND (摘要:('预测模型' OR '风险评估模型' OR '统计模型' OR '临床预测模型' OR '预测模型构建' OR '预测模型验证' OR '预测模型评估' OR '预测模型优化' OR '预测模型应用')  OR 主题词:('预测模型' OR '风险评估模型' OR '临床预测模型')) | 822 |

Supplementary Figure 1. Deeks' funnel plot of the random model meta-analysis of pooled sensitivity, specificity, and area under the curve for the 12 studies.

Supplementary Figure 2. Deeks' funnel plot of the random model meta-analysis of pooled odds ratio for the 7 studies.

Supplementary Figure 3. Subgroup analyses of pulse wave velocity.

Supplementary Figure 4. Subgroup analyses of pattern.

Supplementary Figure 5. Subgroup analyses of pattern unit.

Supplementary Figure 6. Subgroup analyses of qi deficiency syndrome.

Supplementary Figure 7. Subgroup analyses of blood stasis syndrome.

Supplementary Figure 8. Subgroup analyses of yang deficiency syndrome.

Supplementary Figure 9. Subgroup analyses of hypertensive history.

Supplementary Figure 10. Subgroup analyses of diabetes history.

Supplementary Figure 11. Subgroup analyses of smoking history.

Supplementary Figure 12. Subgroup analyses of hyperlipidemia.

Supplementary Figure 13. Subgroup analyses of clinical symptoms of coronary heart disease.

Supplementary Figure 14. Subgroup analyses of clinical symptoms of age.

Supplementary Figure 15. Subgroup analyses of clinical symptoms of sex.
